# *Notes from the Field:* Inspection of 59 “Vape Shops” — United States, October–November, 2016

**DOI:** 10.15585/mmwr.mm6734a6

**Published:** 2018-08-31

**Authors:** David Keith, Kristina Peters, Corinne G. Husten

**Affiliations:** ^1^Office of Compliance and Enforcement, Center for Tobacco Products, Food and Drug Administration, Silver Spring, Maryland; ^2^Office of the Center Director, Center for Tobacco Products, Food and Drug Administration, Silver Spring, Maryland.

In 2009, the Food and Drug Administration (FDA) was given the authority to regulate the manufacture, marketing, and distribution of tobacco products (*1*). Effective August 2016, this authority was extended to all tobacco products, including electronic cigarettes (e-cigarettes) (*2*). Section 704(a)(1) of the Food, Drug, and Cosmetic Act gives FDA authority to conduct investigations and inspections of manufacturers (i.e., any person who manufactures, fabricates, assembles, processes, or labels a tobacco product or imports a finished tobacco product for sale or distribution in the United States) to ensure compliance with applicable federal requirements (*1*,*2*).* In October and November 2016, unannounced inspections were conducted in 59 “vape shops” to learn about business and manufacturing practices, including whether establishments were retailers or manufacturers, and, among manufacturers, how the products sold were manufactured and whether the manufacturers were aware of FDA regulations regarding tobacco products. This report summarizes these first 59 inspections, which showed a lack of quality assurance programs, standard operating procedures, and full labeling of ingredients by the inspected manufacturers.

Vape shops engage in activities that include selling electronic nicotine delivery systems (ENDS), ENDS replacement pieces, and ENDS premixed flavored “e-liquids” and mixing or preparing combinations of liquid nicotine, flavors, and other liquids for sale to consumers. Before an inspection was initiated, a notice of inspection was issued to the most senior staff member at the shop, who also received a summary of the findings once the inspection was closed. Sixty establishments in four states were selected; one smoking lounge that did not sell or manufacture products was excluded from subsequent analysis.

The 59 inspected shops included 31 retailers that only sold finished products, 27 that were both manufacturers and retailers, and one manufacturer that did not sell to consumers. Personnel at all shops reported being aware of FDA tobacco product regulation. The remainder of this report focuses on the 28 manufacturers.

Among the 28 manufacturers, 14 were small businesses employing approximately three persons each; the remaining 14 reported having a parent corporation, a subsidiary, or an affiliate company. All 28 manufacturers sold brands of tobacco products made by other manufacturers as well as house brands manufactured on-site in an area away from customers. No assessed manufacturers allowed customers to mix their own products on-site.

Among the 28 manufacturers inspected, 25 identified a nicotine concentration on the label of products offered for sale, which ranged from 0–100 mg/mL. During the manufacturing process, the assessed manufacturers generally reported using recipes to make products and employed automated pipettes, graduated burettes, or disposable syringes to measure the volumes of liquid, including nicotine, flavoring, propylene glycol, and vegetable glycerin specified in the recipe. Only one manufacturer reported testing the finished products to ensure the product contained the concentration of nicotine indicated on the label. Six establishments indicated that they request testing records from the manufacturers of the branded tobacco products they sold. None of the establishments had quality assurance programs or practices, standard operating procedures, or standardized job training for manufacturing house brands of tobacco products. Workers received on-the-job training and used recipes.

This is the first assessment of business and manufacturing practices at vape shops, which have only recently become regulated. Although all vape shops inspected were found to be in compliance with the regulatory requirements of FDA that were in effect at the time of the inspection, the lack of quality assurance programs, standard operating procedures, and full labeling of ingredients by the inspected manufacturers suggests that consumers might not receive complete information regarding product contents or purchase products of consistent quality; these concerns might be addressed by future FDA regulatory activities. FDA continues to conduct inspections, which will provide additional information on industry practices and compliance with FDA’s regulatory requirements and might inform state and local vape shop policies.

## Footnotes

* https://www.fda.gov/TobaccoProducts/Labeling/default.htm.

## References

[1] Family Smoking Prevention and Tobacco Control and Federal Retirement Reform, Pub L. No. 111–31, 123 Stat. 1776 (June 22, 2009). https://www.gpo.gov/fdsys/pkg/PLAW-111publ31/html/PLAW-111publ31.htm

[2] Food and Drug Administration. Deeming tobacco products to be subject to the Federal Food, Drug, and Cosmetic Act, as amended by the Family Smoking Prevention and Tobacco Control Act; restrictions on the sale and distribution of tobacco products and required warning statements for tobacco products. Final rules. Fed Regist 2016 May 10;81(90):28973–9106. 27192730

